# Pay-it-forward to increase uptake among 15–18-year-old adolescent girls compared with user-paid vaccination: The pilot results of a two-arm randomized controlled trial in China

**DOI:** 10.21203/rs.3.rs-2903998/v1

**Published:** 2023-05-25

**Authors:** Chuanyu Qin, Yifan Li, Shengyue Qiu, Yu He, Vivian Wan-Cheong Yim, Shenglan Tang, Heng Du, Wenfeng Gong, Joseph D Tucker, Weiming Tang, Leesa Lin, Jennifer Smith, Dan Wu, Jing Li

**Affiliations:** Sichuan University No 4 West China Teaching Hospital: Sichuan University West China School of Public Health; Sichuan University No 4 West China Teaching Hospital: Sichuan University West China School of Public Health; Sichuan University No 4 West China Teaching Hospital: Sichuan University West China School of Public Health; Community Health Center; The Chinese University of Hong Kong; Duke Kunshan University; Bill & Melinda Gates Foundation; Bill & Melinda Gates Foundation; London School of Hygiene & Tropical Medicine; The University of North Carolina at Chapel Hill School of Medicine; London School of Hygiene & Tropical Medicine; UNC Gillings School of Global Public Health: The University of North Carolina at Chapel Hill Gillings School of Global Public Health; London School of Hygiene & Tropical Medicine; Sichuan University No 4 West China Teaching Hospital: Sichuan University West China School of Public Health

**Keywords:** human papillomavirus (HPV), vaccination, pay-it-forward, pilot study, feasibility, China

## Abstract

**Background:**

China has low human papillomavirus(HPV) vaccination rate due to lack of public funding and mistrust in domestic vaccines. This pilot study evaluated the feasibility and preliminary effectiveness of an innovative pay-it-forward strategy, which has a participant receive a subsidized vaccine and offers her an opportunity to donate to support other girls, in improving HPV vaccine uptake among 15–18-year-old adolescent girls.

**Methods:**

A two-arm randomized controlled pilot trial was performed in one vaccination clinic in Western China. Adolescent girls (via caregivers) were invited to participate the study via online dissemination of the pilot study. Eligible ones were then randomly allocated using a sealed envelope method into standard-of-care or pay-it-forward arm in a 1:1 ratio. Pay-it-forward participants received hand-written postcard messages, a subsidized vaccine, and an opportunity to donate and (or) write postcards for future recipient girls. Standard-of-care participants self-paid for vaccines. The primary outcome was the first-dose HPV vaccine uptake, which was calculated using multivariable logistic regression, presented as crude/adjusted odds ratios (cORs/aORs) and 95% con dence intervals (CIs). Standard scales were used to assess the feasibility of the program.

**Results:**

A total of 100 participants (50 in each arm) were recruited from Jan 4, 2022 to Feb 18, 2022. The HPV vaccine uptake rate was 98% (49/50) in the pay-it-forward arm and 82% (41/50) in the standard-of-care arm (*c*OR = 10.76, 95% *CI*: 1.31–88.47, *P* = 0.027; a*OR* = 12.12, 95% *CI*: 1.37–107.29, *P* = 0.025). The completion rate of full schedule of HPV vaccination in the two arms was 100% (49/49) and 95.1% (39/41), respectively. Of all 49 vaccinated girls in the pay-it-forward arm, 38 (77.6%) donated to support future participants, and the total donation accounted for 33.3% of prepaid subsidization. Among caregivers in the pay-it-forward arm, 97.6% (41/42) believed that this strategy was feasible.

**Conclusions:**

The pilot showed feasibility and preliminary effectiveness of a pay-it-forward strategy to increase HPV vaccination rate. The high uptake rate in the standard-of-care arm is likely caused by the selection bias associated with the online dissemination approach and secured vaccine supply through the program. Further adaption of the intervention package and a population-based recruitment pathway are needed to better reflect local contexts and enhance the generalizability of the subsequent formal trial.

**Trial registration:**

Chinese Clinical Trial Registry (ChiCTR), ChiCTR2200055542. Retrospectively registered on 11 January 2022, https://www.chictr.org.cn/showproj.html?proj=139738.

## Background

Cervical cancer is the most common cancer affecting women’s health worldwide, and is the fourth leading cause of cancer death among women [1]. Over 341 thousand women died from cervical cancer worldwide in 2020, and more than 15% occurred in China [1, 2]. And within China, cervical cancer mortality is higher in the west-central regions than that in the eastern regions, and it is also higher in the rural than that in the urban areas [3].

HPV vaccine is the most cost-effective preventive measure [4]. Currently, five HPV vaccines (three imported vaccines Cervarix ^®^, Gardasil^®^ and Gardasil 9^®^ and two domestic vaccines Cecolin^®^ and Walrinvax^®^) have been approved in China. But the HPV vaccination rate is low in the country. In 2019, among females aged 15–24 years, only 11% of them were vaccinated against HPV, and Western China had the lowest vaccination rate at 8.6% in comparison to Central (10.6%) and Eastern China (13.7%)[5]. High costs of vaccines, lack of public funding, inadequate supply of the imported nonvalent HPV (9v-HPV) vaccine, and mistrust in domestic vaccines are major reasons [6–8].

The World health Organization (WHO) recommends to include HPV vaccine in national immunization program and suggested priority populations for vaccination [9]. Up to date, 125 countries and regions have introduced HPV vaccine into the national immunization program. In recent years, several Chinese municipal governments started to pilot public immunization programs for girls aged 13–14 years [10–13]. However, these programs left an important catch-up age arm (15–18 years) behind. This is an age range when adolescents start to explore sexuality and the protective efficacy of HPV vaccination may rapidly decline due to the risks of exposure to HPV viruses and immunogenic responses to vaccination [14, 15]. Evidence showed that the average age of the first sexual intercourse among Chinese youth was before the age of 18 [16], suggesting an urgent need of innovative strategies to address these issues to improve coverage among girls aged below 18.

The pay-it-forward innovation may be such a solution. Pay-it-forward has a girl receive community supported and subsidized HPV vaccination and then offers an opportunity for her to donate/write postcard messages to encourage other girls to vaccinate. Our previous studies have shown that pay-it-forward effectively increased service uptake, including two trials focused on gonorrhoea and chlamydia testing and one trial on in influenza vaccination [17, 18]. However, the applicability of pay-it-forward is unknown in promoting HPV vaccination for another population with substantially different contextual backgrounds, which providing an idea on improving HPV vaccine uptake among girls of appropriate age. This pilot of a two-arm randomization-controlled trial (RCT) aimed to examine the feasibility and preliminary effectiveness of the pay-it-forward strategy. The pilot data helped inform the re refinement of the intervention package and implementation process in vaccination clinics for the two-arm RCT.

## Methods

### Study design and settings

The two-arm RCT compared the effectiveness of a pay-it-forward strategy against the standard-of-care approach in increasing HPV vaccination among 15–18-year-old adolescent girls. The pilot was conducted in Yulin Community Health Center (CHC) in the downtown area of Chengdu, Western China. CHCs in China are the urban primary care system that provides essential medical care, preventive services, and routine health promotion and management for local residents [19]. Given the strict coronavirus disease 2019 (COVID-19) control measures and heavy clinic workload, we discussed with the CHC collaborator and agreed upon two possible recruitment pathways for the pilot. One was setting up a convenient recruitment site in a public space nearby the vaccination clinic. However this approach turned out to be inefficient in identifying eligible caregivers and girls due to limited pedestrian traffic during the COVID control periods and required extra administration efforts, and hence was unfavorable by our clinic staff. The other approach was online recruitment via disseminating information about the pilot through the CHC’s social media public account [20]. This method was more acceptable to the clinic staff as it is the conventional way the CHC promotes vaccine services to local residents. The pilot was then implemented from Jan 4, 2022, to Feb 18, 2022.

### Participants

Participants were adolescent girls living in the communities served by Yulin CHC. The eligibility criteria were: 1) aged between 15–18 years, 2) no HPV vaccination history, 3) no prior allergies to vaccination; and 4) have a legal guardian consenting to participate the study. Exclusion criteria included: 1) age below 15 or above 18 years, 2) received HPV vaccination, 3) risk of allergy to HPV vaccines by clinical assessment, or 4) refused to consent to participate. Participant eligibility was screened by a research assistant (RA).

### Enrollment, randomization and blinding

Eligibility of participants who came to the clinic after the online promotion was screened and verified by RAs. They introduced the program and obtained written informed consent from caregivers and girls who agreed to participate. Eligible participants were randomly assigned to either the standard-of-care arm or the pay-it-forward arm at a ratio of 1:1 using a sealed envelope method. Individual randomization involved two steps. First, 100 random digits were generated by SPSS (version 21.0) and sorted from smallest to largest, the smaller 50 digits were assigned as the control arm and the larger 50 digits were assigned as the pay-it-forward arm. These digits along with assigned intervention materials were assembled into envelopes. Second, each enrolled participant drew an envelope in a sequential order on a temporal basis and was assigned to either of the arms. Different RAs assembled the envelopes and conducted assignment independently. All staff were unaware of which treatment one individual participant was receiving until the participant opened the envelope. The recruiting RA then guided participants to a private room according to the assigned arm for intervention delivery which was conducted by other RAs. Assignment and interventions delivered to participants were blinded to vaccination personnel in the clinic and data analysts. However, it was impossible to completely blind the treatment one participant received to the RAs who delivered the intervention due to the nature of a behavioral intervention. But since the assignment and delivery procedures were independently done by different people, it was impossible for the two assistants who delivered interventions to determine or alter the possibility of an individual to receive a treatment.

## Procedures

### Interventions and consents

An educational pamphlet that provided a brief introduction to cervical cancer, HPV vaccination, types and costs of HPV vaccine products was given to all participants in both two arms. This educational pamphlet is provided in routine practices by CHCs and details can be found in Additional le 1. All participants completed a paper questionnaire survey (Additional file 2) immediately after receiving the intervention.

### Pay-it-forward intervention

Participants in the pay-it-forward arm were told the market prices of various HPV vaccines and that previous participants donated to support their vaccination (a subsidy of US$ 149.8 per person). If they decided to receive the subsidy and a vaccination, they were then also given an opportunity to donate to support more future participants. The donation was voluntary and anonymous, and donation decisions did not affect their receiving a subsidy nor any other services at the clinic. Besides, participants were shown handwritten postcard messages and invited to create their own messages (Additional file 3: p 1–3).

### Standard-of-care intervention

Participants in the standard-of-care arm needed to pay out of pocket at the standard market price if they decided to receive HPV vaccination. However, they didn’t receive community-engaged messages or information relevant to pay-it-forward.

### Vaccination and donation

Girls (via caregivers) who decided to vaccinate made an appointment to visit the clinic again within two weeks for administration of vaccination. Clinical staff assessed clinical eligibility again for receiving an HPV vaccine. We followed up with all participants who booked an appointment and collected data on the first-shot, second-shot and full schedule of vaccination 6 months after intervention delivery. After vaccination and while they waited, girls and/or their caregivers from the pay-it-forward arm were provided with an opportunity to contribute, monetarily and/or non-monetarily by sending a warm message in a designed postcard (Additional file 3: p4–6) to help future girls to receive HPV vaccination.

### Outcome measures

The primary outcome was the first-dose HPV vaccine uptake in two arms. The secondary outcomes were the completion of the full schedule (3-doses) of HPV vaccination in both arms and the donation amount from the pay-it-forward arm. The number of participants who received at least one shot and their choices of vaccines were also analyzed. Paper-based questionnaires were used to collect data on a): sociodemographic characteristics; b): willingness to be vaccinated and its related reasons; c) their donation behavior after the receipt of the first-dose (pay-it-forward arm only). Electronic questionnaires were also implemented to investigate parental attitudes towards their preference to the types of HPV vaccines. The acceptability, appropriateness and feasibility were evaluated by using the standard Acceptability of Intervention Measure (AIM), Intervention Appropriateness Measure (IAM), and Feasibility of Intervention Measure (FIM) Scales (Additional file 4) that have been well evaluated before[21]. Each scale has 4 items and every item has five different options, ranging from 0 for completely disagree to 5 for completely agree. The higher the score is, the greater acceptability, appropriateness, and feasibility of pay-it-forward strategy that were achieved.

Two trained master students were recruited to double-enter data from the paper to computer-based database (EpiData) independently. All finished double-entry databases were validated by running EpiData (version 3.1). Any inconsistency found between the two databases was adjusted and corrected until the databases agreed. As final check, one of databases was chosen and underwent a final consistency check and then was finalized for analysis.

### Statistical analysis

No power analysis was performed to estimate the sample size as for a pilot study to evaluate the feasibility and potential effectiveness of a pay-it-forward strategy [22]. Quantitative data was calculated and presented by means and standard deviations (SDs). Descriptive statistics were used to analyze data for a single categorical variable include frequencies and percentages. Chi-squared tests (χ^2^ tests) and Student t-tests were used to compare the characteristics and outcomes between two arms (the standard of care vs. pay-it-forward). Multivariable logistic regression was used to calculate odds ratios (*OR*s) and 95% confidence intervals (95% *CI*s) adjusted for relevant parameters (e.g. parents’ gender, education, and family income) as identified in preliminary data analysis. SPSS Statistics (version 21.0) and Microsoft Excel 2010 were used to analyze the data. Statistical significance was assessed by two-tailed tests with level of 0.05.

## Results

### Characteristics of caregivers between the pay-it-forward and the standard-of-care arm

**Figure 1:**
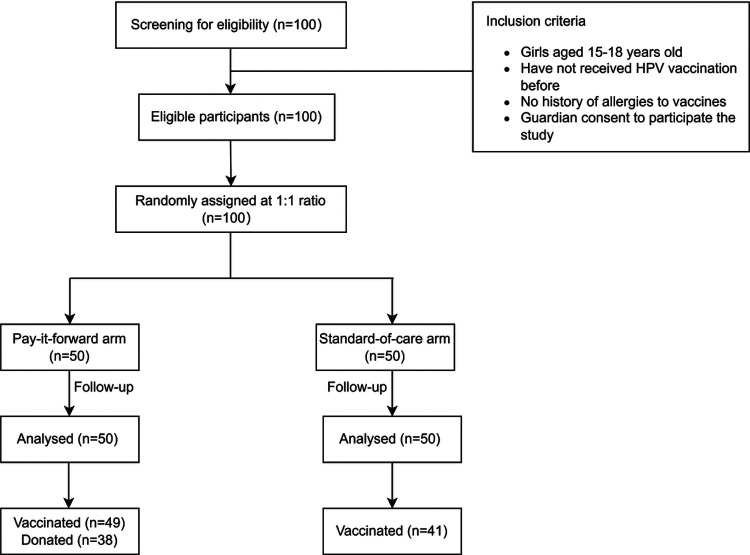
Study flow chart.

A total of 100 participants were included for final analysis (Fig. 1). The mean age of caregivers was 45.6 years (SD = 5.6), with the majority (84.0%) being females. Up to 70% had college or higher education, and 74% had an annual income ranging from $4539 to $22693. No difference was seen between the two arms regarding the caregivers’ demographic characteristics (Table 1).

### The first-dose HPV vaccine uptake and the completion of the full schedule of HPV vaccination in both arms

**Figure 2:**
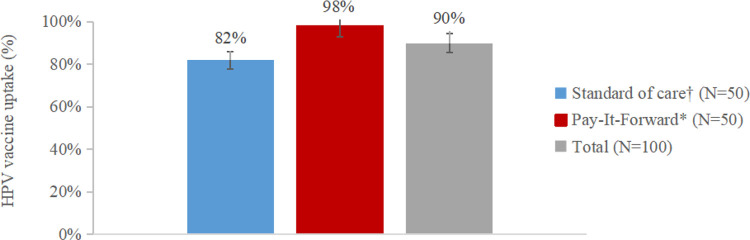
HPV vaccine uptake rates by study arms. †Standard-of-care refers to self-paid vaccination. *Pay-it-forward refers to the community-based intervention that offered a subsidized vaccine with US$149.8 and participants could choose to donate to support more people.

Overall, 90% girls received vaccination. Among which, 49 were in the pay-it-forward arm and 41 were in the standard-of-care arm. The vaccine uptake was 98% (49/50) in the pay-it-forward arm vs. 82% (41/50) in the standard-of-care arm (Fig. 2). Compared with the standard-of-care arm, the pay-it-forward arm was associated with a 16% absolute increase in the proportion of participants who got vaccinated (*cOR* = 10.76, 95%*CI*: 1.31–88.47, P = 0.027, Additional file 5). Adjusted for parents’ gender, education, and family income, we found that the health behavior of receiving first-dose HPV vaccination in the pay-it-forward arm was 12.12 times of that in the standard-of-care arm (95%*CI*: 1.37–107.29, *P* = 0.025, Additional file 5). The completion of full schedule HPV vaccination in the pay-it-forward arm was 100%, however, it was 95.1% in the control arm (Table 2).

### Choices of vaccines and parental preference to the types of HPV vaccines

Except for 5 girls who chose 2v-HPV vaccine because they were ineligible for the 9v-HPV vaccine due to the indicated age from the instruction, 98% (48/49) in the pay-it-forward arm and 90% (37/41) in the control arm chose 9v-HPV vaccines. The choice of vaccine types was generally consistent in both arms (Table 2). When asked about what would they do if the 9v-HPV vaccine was out-of-stock at the CHC, 71% (64/90) caregivers would like to wait to vaccinate their daughter until the 9v-HPV vaccine becomes available. No difference was observed between arms (Table 2).

### Willingness to be vaccinated and related reasons

The primary reason for caregivers to vaccinate their daughters was that HPV vaccine could protect them from developing cervical cancer (45 replies in the pay-it-forward arm and 34 replies in the standard-of-care arm, Table 2). The secondary reason was that they had plans for HPV vaccination (37 replies in the pay-it-forward arm and 33 replies in the standard-of-care arm, Table 2).

### Donation behavior and donation amount

**Figure 3:**
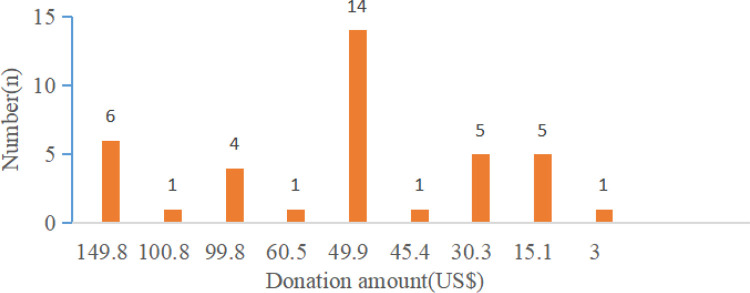
Donation distribution in the pay-it-forward arm. The number of participants that make a donation and the corresponding donation amount in the pay-it-forward arm.

Of the 49 participants, 38 (77.6%) actually contributed some amount through the pay-it-forward program, and 26 (68.4%) donated $49.9 and above. The mean donation per person was $69.4 (SD = 49.9). The largest donation was $149.8 and the median donation was about $49.9 (IQR 69.6). The total amount of donations ($2433.6) accounted for 33% of the prepaid fees ($7488.7) and could cover 16 individuals in the future for HPV vaccination (Fig. 3).

### Acceptability, appropriateness, and feasibility assessment

Out of 50 participants in the pay-it-forward arm, we obtained responses from 42 individuals. Thirty-eight (90.5%), 41(97.6%) and 41(97.6%) of 42 participants believed that the pay-it-forward strategy was acceptable, appropriate, and feasible, respectively. The scale scores for acceptability, appropriateness and feasibility were 18.57(SD = 3.04), 18.81(SD = 1.84), and 18.71(SD = 1.92), respectively. More than 95% of the participants had consent or full consent attitudes (Table 3).

## Discussion

For the first time, our study contributes to the literature by assessing the feasibility, acceptability, appropriateness and preliminary effectiveness of a social innovation intervention using a two-arm RCT design, developing new methods for HPV vaccination engagement among adolescent girls, and enhancing vaccine uptake. Our findings suggest that the pay-it-forward strategy is feasible, acceptable and appropriate to implement among the target population. This strategy might increase HPV vaccine uptake among the 15–18 adolescent girls compared with a standard-of-care strategy for vaccination in China. This strategy increased vaccine uptake, elicited financial contribution, improved the public engagement and was correlated with vaccine confidence.

The finding that those who took part in the pay-it-forward intervention had higher HPV vaccine uptake than those in the standard-of-care arm is consistent with previous intervention studies using pay-it-forward interventions to improve health services uptake and influenza vaccine uptake [17, 18, 23]. The vaccination uptake in both arms were significantly higher than that (11.0%) in the previous study among women aged 15–24 years in western China in 2019 [5]. The vaccination rate in the pay-it-forward arm was also higher than the rate in Chinese cities that were selected as demonstration sites to fully or partially subsidize HPV vaccines among 13–14 years old adolescent girls [10–12, 24, 25]. e.g., in Chengdu, the HPV vaccinate uptake was around 90% in the government sponsored program since its implementation in year 2022 according to the unpublished data. Similar to the influenza vaccine study [18], the effect of pay-it-forward interventions might be related to the subsidized/reduced costs of vaccination, enhanced community engagement, vaccine confidence, or a combination of these. However, compared with influenza vaccine, HPV vaccines are more expensive with the price varies from ($ 49.8)/dose to ($ 201.1)/dose depending on its valent and supplier; also, a reservation process to get HPV vaccination is required due to the huge demanding vs. limited supplies, which poses a more complicated contextual background to evaluate the effectiveness of pay-it-forward strategy in increasing the HPV vaccine uptake. In this pilot study, the uptake in both arms is high, indicating that the waived waiting time/appointment (several months to two years) to get the 9v-HPV vaccines that were always unavailable in the daily clinical practice might be a great contribution (confounder) as well.

We also observed that, among those enrolled in the pay-it-forward arm, the majority of them (77.6%) voluntarily donated to support another girl after receiving an HPV vaccine. The average donation was $69.4 and the total donation could cover 33% of prepaid fees which was similarly the previous studies [17, 23]. The willingness to donate and the amount were determined by the caregivers and the girls after joint discussion, except for a small number of girls who went to vaccination alone because they were 18 years of age. Donations collected using a pay-it-forward system can support more individuals in receiving HPV vaccine services, which could be an important social innovation for improving HPV vaccine uptake when government-funded vaccination is unavailable or as an important supplementary approach to help a different appropriate age group when government-funded vaccination is available. Pay-it-forward interventions could help transition out-of-pocket payments to government-funded HPV vaccine programs. Furthermore, the possibility of creating an urban-to-rural subsidization mechanism to support HPV vaccination in poorer areas should also been taken into consideration.

Pay-it-forward interventions have additional social benefits, fostering community engagement [18, 26]. Community engagement is central to the success of public health programs. Engaging the community in vaccination services through cultivating kindness and reciprocity might also strengthen community solidarity and increase confidence in vaccine services [27, 28]. In this pilot, we encouraged girls who received the first-dose to write some warm kindness for the future girls who would receive HPV vaccines on a predesigned postcard (Additional file 3: p 4–6) by a college student in our research arm. Among the 50 girls from the pay-it-forward arm, 21 had written words on the postcards.

The findings observed suggested that parents in both arms preferred to the 9v-HPV vaccines even when the 9v-HPV vaccines were unavailable or their daughters were ineligible to get the 9v-HPV vaccines in mainland China. This phenomenon is representative of the general public especially among the metropolitan areas that the choose of HPV vaccines is based on the misleading principle that “the more valent, the better” rather than “the earlier, the better”. As evidence has shown that 87% of cervical cancer and 97% of cervical intraepithelial neoplasia grade 3 (CIN3) could be prevented when administrated at an early age (12–13 years) even with the bivalent HPV (2v-HPV) vaccines [29–31]. This finding highly recommended that health campaigns, educations through different ways should be engaged to increase the awareness and correct knowledge of the general public on HPV vaccines about “the earlier, the better” and “not to be infected when waiting for the 9v-HPV vaccines”. It also hints us that in our future study, more evidence-based information on HPV vaccines should be added in the design of postcards and to educate the caregivers and their daughters when they participate in our future formal study.

The study has several limitations that should be considered and revised for the formal study. First, an easy sampling through online recruitment was conducted because it was implemented during the COVID-19 pandemic period. People were inconvenient to go outside because of the lock-downs. Although the online recruitment was an easy way to recruit a sufficient number of eligible participants within a short time, it led to selection bias. As those who paid attention on the posts and advertisements from the local CHC were well educated and had higher annul income as well as higher health concerns on themselves and their family members and that’s why we see HPV vaccine uptake was high in both arms in this pilot study. Thus, randomization based on the residency list to recruit is highly recommended. The protocol was revised accordingly as well. Second, the high uptake might due to the availability and accessibility of the 9v-HPV vaccines as well as the waive of its waiting time/appointment, as even up to date, the 9v-HPV vaccines are in huge demand and the average waiting time was from several months to 2 years. This might be an important confounding factor that affected our study result. To avoid this confounding in our formal RCT, we will need to revise the protocol from to reserve all kinds of HPV vaccines on purpose for our study to only provide the participants with any kind of HPV vaccine that was in full stock during their routine clinical practice.

## Conclusions

In conclusion, the pilot RCT showed that pay-it-forward strategy was feasible, acceptable and appropriate to implement among adolescent girls aged 15–18 years of age. It had potential to improve HPV vaccination rate among girls of appropriate age, although conclusive closure cannot be made due to the limited sample size, locations and important selection bias and confounders found. The pilot data helped inform the refinement of the intervention package and implementation process in vaccination clinics for the formal two-arm RCT with multi-center and expanded sample size.

## Figures and Tables

**Table 1:** Demographic characteristics of caregivers by arms.

	Total (N = 100)	Pay-it-forward (n = 50)	Standard-of-care (n = 50)	*P*-value†
Mean age, years (SD)	45.6(5.6)	45.7(7.1)	45.6(3.6)	0.920
Gender				0.275
Male	16(16.0)	6(12.0)	10(20.0)	
Female	84(84.0)	44(88.0)	40(80.0)	
Marital status				0.108
Unmarried	3(3.0)	3(6.0)	0(0)	
Married	90(90.0)	42(84.0)	48(96.0)	
Divorced	7(7.0)	5(10.0)	2(4.0)	
Education				0.081
Below college	30(30.0)	11 (22.0)	19(38.0)	
College and above	70(70.0)	39(78.0)	31 (62.0)	
Annual income (US$)				0.832
4539	20(20.0)	8(16.0)	12(24.0)	
4539–12103	22(22.0)	11 (22.0)	11 (22.0)	
12104–22693	32(32.0)	18(36.0)	14(28.0)	
22694–45386	17(17.0)	9(18.0)	8(16.0)	
45386	9(9.0)	4(8.0)	5(10.0)	
Relationship				0.084
Father	14(14.0)	4(8.0)	10(20.0)	
Mother	80(80.0)	41 (82.0)	39(78.0)	
Others‡	6(6.0)	5(10.0)	1 (2.0)	

Data were n (%), mean (SD), unless otherwise specified.

† Student t-tests for continuous data and χ2 tests for categorical data.

‡ Legal caregivers other than parents.

**Table 2:** Information about vaccination in both arms.

		Pay-it-forward (n = 49)	Standard-of-care (n = 41)	Total (N = 90)
The completion rate of HPV vaccination	2-dose	49(100.0)	41/41(100.0)	90/90(100.0)
	3-dose	49(100.0)	39/41(95.1)	88/90(97.8)
Selection of HPV vaccine types	2v-HPV vaccine	1/49(2.0)	4/41(9.8)	5/90(5.6)
	9v-HPV vaccine	48/49(98.0)	37/41(91.2)	85/90(94.4)
Reasons for HPV vaccination*	a. Having plans for HPV vaccination	37/49(75.5)	33/41(80.5)	70/90(77.8)
	b. Could protect me from developing cervical cancer	45/49(91.8)	34/41 (82.9)	79/90(87.8)
	c. Advice from Friends / family	18/49(36.7)	10/41 (24.4)	28/90(31.1)
	d. Suggestions from the medical staff	16/49(32.7)	8/41(19.5)	24/90(26.7)
	e. pay-it-forward program	14/49(28.6)	0/41(0)	14/90(15.6)
Parental preference towards types of HPV vaccines	a. Opting for 4v-HPV vaccine	7/45(16.0)	5/45(11.0)	12/90(13.0)
	b. Opting for domestic 2v-HPV vaccine	1/45(2.0)	4/45(9.0)	5/90(6.0)
	c. Opting for imported 2v-HPV vaccine	2/45(4.0)	1/45(2.0)	3/90(3.0)
	d. Not receive a vaccine now but wait until there are 9v-HPV vaccines	32/45(71.0)	32/45(71.0)	64/90(71.0)
	e. Look out for other channels	3/45(7.0)	3/45(7.0)	6/90(7.0)

Data were n (%).

* The sum of the numbers for each reason was higher than that of people who vaccinated in each arm because there was more than one reason for HPV vaccination.

**Table 3:** Acceptability, appropriateness, and feasibility assessment in the pay-it-forward arm.

Dimension	Item	Attitude*(N = 42)			Dimension scores (SD)	Scoring at 16 and above†
		Completely disagree/Disagree	Neither agree nor disagree	Agree/Completely agree		
AIM	a. It meets my approval.	1(2.4)	1 (2.4)	40(95.2)	18.6(3.0)	38(90.5)
	b. It is appealing to me.	1(2.4)	2(4.8)	39(92.9)		
	c. I like it.	1(2.4)	2(4.8)	39(92.9)		
	d. I welcome it.	1(2.4)	1(2.4)	40(95.2)		
IAM	a. It seems fitting.	0(0)	1(2.4)	41 (97.6)	18.8(1.8)	41(97.6)
	b. It seems suitable.	0(0)	0(0)	42(100.0)		
	c. It seems applicable.	0(0)	0(0)	42(100.0)		
	d. It seems like a good match.	0(0)	1(2.4)	41 (97.6)		
FIM	a. It seems implementable.	0(0)	0(0)	42(100.0)	18.7(1.9)	41(97.6)
	b. It seems possible.	0(0)	1(2.4)	41 (97.6)		
	c. It seems doable.	0(0)	1(2.4)	41 (97.6)		
	d. It seems easy to use.	0(0)	2(4.8)	40(95.2)		

Data were n (%), mean (SD).

* Number of people with a different attitude for each item.

† Number of people scoring at 16 and above for each scale.

## Data Availability

The datasets used and/or analysed during the current study are available from the corresponding author on reasonable request.

## Abbreviations

HPV: Human papillomavirus
c*OR*s: Crude odds ratios
a*OR*s: Adjusted odds ratios
*CI*s: Confidence intervals
9v-HPV: nonvalent HPV
WHO: World health organization
RCT: Randomized controlled trial
CHC: Community Health Center
COVID-19: Corona Virus Disease 2019
RA: Research assistant
AIM: Acceptability of Intervention Measure
IAM: Intervention Appropriateness Measure
FIM: Feasibility of Intervention Measure
SDs: Standard deviations
χ^2^ tests: Chi-squared tests
*OR*s: Odds ratios
CIN3: Cervical intraepithelial neoplasia grade 3
2v-HPV: Bivalent HPV
